# Isocyanic acid measurements in Swedish industries and environments

**DOI:** 10.1007/s11356-026-38302-w

**Published:** 2026-10-15

**Authors:** Gunilla Runström Eden, Tobias Storsjö, Anders Johansson, Lena Andersson, Håkan Tinnerberg

**Affiliations:** 1 Occupational and Environmental Medicine, Sahlgrenska University Hospital, Gothenburg, Sweden; 2 Occupational and Environmental Medicine, School of Public Health and Community Medicine, Institute of Medicine, University of Gothenburg, Gothenburg, Sweden; 3 Occupational and Environmental Medicine, Örebro University Hospital, Örebro, Sweden; 4 Occupational and Environmental Medicine, Faculty of Medicine and Health, Örebro University, Örebro, Sweden

**Keywords:** Isocyanates, Occupational exposure, Air pollutants, Environmental monitoring, Polyurethanes, Urea chemistry

## Abstract

Isocyanic acid (ICA), the simplest monoisocyanate, is formed mainly as a by-product through thermal degradation of nitrogen-containing materials. Although ICA participates in protein carbamoylation, human health data remain limited. Existing studies have shown that certain industrial processes may generate substantial ICA emissions but other occupational settings have not been studied. Environmental concentrations are lower than industrial but have not been studied in Europe. This study aimed to survey ICA concentrations in a wide range of occupational and environmental settings in Sweden. In total, 173 air samples were collected in 16 environments. Both stationary and personal sampling were performed. Concentrations were compared to the only existing occupational exposure limit (OEL, 18 µg/m^3^ 8 h mean) to aid interpretation. High ICA concentrations were consistently observed in industries using urea-based binders at elevated temperatures, with maximum stationary levels exceeding 250 µg/m^3^. Newly installed mineral wool insulation during initial heating phases generated elevated emissions up to 64 µg/m^3^. Thermal processing of polyurethane produced lower but spatially widespread concentrations throughout large work areas. In contrast, ICA levels in newly investigated environments—including restaurants, food industries, linoleum welding, diathermy procedures, asphalt work and lawn mowing—were generally well below OEL or below detection limits. Outdoor air measurements in Gothenburg, Sweden, showed uniformly low concentrations (mean 0.12 µg/m^3^). Extremely high ICA levels were confirmed in direct cigarette smoke (4300 µg/m^3^), while side-stream exposure was considerably lower. Overall, the survey shows that higher ICA concentrations are mainly associated with industrial high-temperature handling of urea- or polyurethane-based materials, as well as cigarette smoke. Other investigated occupational settings showed low or negligible ICA levels under the conditions measured.

## Introduction

Isocyanic acid (ICA) is the simplest monoisocyanate, with the structural formula HNCO, and has been known for a long time but has, until recently, received limited interest from researchers. ICA is not registered as a commercial substance by REACH and is mostly present as a by-product from different thermal processes. Combustion is a common source for isocyanic acid and emissions have been shown from cigarette smoke (Runström Eden et al. 2025, Baker and Bishop 2004, Hems et al. 2019), biomass burning (Leslie et al. 2019) and diesel and gasoline engines (Wentzell et al. 2013; Brady et al. 2014). Measured levels of ICA outdoors are reported to be in the range of 0.018–1.8 µg/m^3^ (Leslie et al. 2019) and indoor levels are reported to be slightly higher but depending on activities inside (Wang et al. 2022). ICA has also been discussed in several other different research fields such as astronomy (Hernandez-Gomez et al. 2019), vehicle catalysis efficiency (Czekaj et al. 2009), biochemistry (Delanghe et al. 2017), indoor air (Nazaroff and Weschler 2020) and lastly occupational medicine (Löfstedt et al. 2009, Lofstedt et al. 2011a; Löfstedt et al. 2011a, b b). Thermal decomposition of polyurethane (PUR) has shown exposure to ICA reaching concentrations of 900 µg/m^3^ (Karlsson et al. 2002); the same can be said for thermal decomposition of formaldehyde-urea resin with reported maximum concentrations reaching 190 µg/m^3^ (Westberg et al. 2005) and 573 µg/m^3^ (Jankowski et al. 2017).

A reason for the increased interest in environmental research is likely due to ICA being part of the isocyanate family, which is well known for giving asthma and other health effects from the respiratory airways (Dalböge et al. 2023). In occupational settings, mainly diisocyanates are studied, but the exposure and health effects after exposure to monoisocyantes are also well known with the tragic disaster in Bhopal as the major source of knowledge (Bucher 1987; Dhara and Dhara 2002), where 15,000 to 20,000 deaths occurred due to the exposure of methyl isocyanate. The knowledge of health effects after exposure to ICA is on the other hand not well studied. It is known that ICA is biochemically active as it has a part in a protein modification called carbamylation (Jaisson et al. 2018). From occupational settings, only one study investigating health effects of monoisocyanates has been identified (Löfstedt et al. 2009; Löfstedt et al. 2011a, b). In this, nasal symptoms and a small reduction in forced expiratory volume in 1 s were seen. The workers in that study had a mixed exposure of monoisocyanates, formaldehyde and dust. In a more recent study of Leandersson (Leandersson and Krapi 2019), three workers exposed to decomposition products emitted during hot iron cauterisation, including levels of ICA (personal measurements between 331 and 435 µg/m^3^), were medically examined. Two of the three had asthma and the third had severe airway problems although it is not clear whether this was linked to the exposure to ICA. Sweden is the only country in the world that has established an occupational exposure limit (OEL) for ICA (18 µg/m^3^ for 8 h and 36 µg/m^3^ for a 5-min period). Corresponding values in ppb are ca 10 and 20 ppb respectively. Health effects data on ICA are scarce; the OEL is based on the similarity to methyl isocyanate (Work and Health Scientific serial 2002).

There are a few studies presenting emissions or exposure in occupational settings and the levels are very much higher than in the environment. Environmental measurements have been performed in the USA but not in the European setting. The present study was designed to expand on the existing knowledge of ICA exposure to gain a comprehensive overview of concentrations in various environments as there might be health implications and European data are lacking. Leandersson et al. (2019) found emissions of ICA while frying common food in a kitchen pan (11.7–37.6 µg/m^3^) which indicated that thermal decomposition of other nitrogen-containing substances could emit ICA and the occupational exposure could be more widespread than previously known. The aim of the study was therefore to perform a survey of ICA emission and exposure levels in different occupational and environmental settings.

## Material and methods

### Recruitment of participating companies

The recruitment strategy was to include both work environments where high exposure could be expected from earlier measurements, to assess the current situation, and new work environments where measurements had not been previously made. Companies were contacted through branch organisations, previous industrial contacts or direct targeted recruitment, based on sector. For known environments, two business organisations, Swedisol and Byggmaterialindustrierna, were contacted. Swedisol is a branch organisation for producers of mineral wool and Byggmaterialindustrierna is an umbrella organisation for companies and other organisations producing or using building materials. Via these organisations, companies producing building materials with high nitrogen content or users of such products were contacted.

### New occupational environments

One of the triggers for the study was the emissions of ICA measured during the pan frying of food (Leandersson 2019). Therefore, several restaurants and food industries were recruited in Gothenburg and Örebro, Sweden. Another occupational setting with potentially high number of exposed workers is in medical care where biological tissue is thermally treated. With this in mind, emissions and exposure during two different diathermy sessions at the University Hospital in Örebro were also included. Floor laying was also identified as a potential work environment with ICA exposure during linoleum mat welding.

Previously identified occupational environments with potentially high exposures of ICA:

It is known that monoisocyanates can be emitted when working with formaldehyde-urea resins at high temperatures (ca 133 °C and above for ICA, Jankowski et al. 2016) and that this kind of material is common in building materials. Companies working with materials using an urea binder and companies employing building insulators were recruited to assess this. Companies where thermal decomposition of polyurethane occurred were also recruited.

### Environmental sources

In addition to traditional occupational exposure, environmental sources were also included. A known source of ICA is cigarette smoke (Runström Eden et al. 2025, Baker et al. 2004). An experimental setup was used to estimate the exposure for a home health care provider if the patients smoke. Another experimental setup was used to study emissions from diesel engines, to compare with environmental concentrations. Environmental research has identified the presence of ICA practically everywhere (Leslie et al. 2019) but there is a lack of data from European environments. To determine this, measurements in urban air and also during use of gasoline-powered lawn mower were performed.

### Collection of air samples

All measurements have been performed after derivatisation during sampling with di-n-butylamine and analysis with liquid chromatography mass spectrometry (LC-MS). The majority of measurements have been performed with the Supelco Easysampler denuder (ASSET EZ4-ICA, Merck, Darmstadt Germany) (Marand et al. 2005). On a few occasions impingers were used, as described by Karlsson et al. (2001) as they allow a higher flowrate, which also decreases the limit of detection, which can be of importance for very short tasks. This was important for urea chip board measurements, which was a very short task. For cigarette smoking, impingers allowed a higher flowrate, which better mimicked the act of smoking a cigarette. Direct cigarette smoke was consequently measured using both impingers and denuders. In each setup, two samplers were coupled together in series (to catch possible breakthrough) which was then directly attached to the cigarette (according to the method described in Runström Eden et al. 2025).

Impingers contained a dibutylamine solution and a backup filter and air was drawn through both in sequence to capture both the gaseous and the particulate fraction of the emission. During personal sampling, the samplers were placed in the breathing zone of the person and worn for the duration of the task. The impinger was placed in a pocket in the breathing zone of the person and was fastened using tape.

For the outdoor measurements, a modified denuder method with a lower air flow was used, which made it possible to measure for a prolonged time as it is known that the background concentration of ICA on the sampler increases with time (Jankowski et al. 2017). The Easysampler air flow was therefore set to 200 ml/min, except for the measurements in the urban air, when we used an air flow ~400 ml/min. When using the impinger, the airflow was set to 1 l/min. All air flows were set with TSI digital flow meter model 4140 (TSI Incorporated, Shoreview, MN, USA) and checked before, during and after the measurements. During all measurements field blank samples were also collected and the achieved measurement results were corrected with the accompanying blanks. Field blanks were treated the same as the actual samples but no air was drawn through them.

### Chemical analysis

Chemical analyses have been performed by the Occupational and Environmental Medicine laboratory in Örebro, Örebro University Hospital Sweden, except the measurements in urban air, which were performed at EHS analytics AB (Staffanstorp, Sweden). Analysis at EHS analytics was performed by LC-MSMS and analysis at the laboratory in Örebro was performed by HPLC–MS/MS (UPLC Accela 1250 with Accela Autosampler and TSQ Quantum Access Max with HESI-II detector). The dibutylamine solution and the impinger filter were analysed separately according to ISO 17734-1:2013. The chemicals used in the analyses were methanol, toluene and 5-mM sulfuric acid for sample preparation. A deuterated internal standard of the analysed isocyanates in acetone was added and the mobile phase used consisted of acetonitrile, formic acid and water. Corresponding LOD and LOQ for the analytical methods were 0.006 µg/sample and 0.020 µg/sample respectively and have been determined according to guidelines from Eurachem 2014. Blank samples from different lots have been analysed repeated times for the determination of LOD and LOQ.

### Measurement strategy

The measurement strategy was devised to determine emissions of and/or exposure to ICA in different environments. All measurements were conducted during the period of June 2020 to September 2021. Personal measurements to measure exposure were used where possible but unfortunately, the COVID-19 pandemic restricted this in many work places and only stationary measurements were then used instead. The number of occupational samples varied depending on the size of the operation and numbers employed in exposed tasks. In a few instances, there were parallel ongoing projects to determine emissions or work place exposures during certain tasks. The present study was able to attach itself to these projects and this allowed for a larger sample set from these environments (manufacturers using urea binder, new insulation and diesel exhaust).

The focus was otherwise to survey as many different environments as possible, rather than performing a traditional exposure assessment, which was planned for a follow-up study in select environments. Due to this, and for practical reasons (e.g. sometimes the emission generating task was only 15 min long), sampling times varied between the different work environments. For environmental measurements of urban air, most sampling times were 20+ h to achieve a quantifiable result as concentrations were expected to be low based on the available literature. The majority of samples were collected in one visit but for manufacturing of urea and diesel exhaust measurements, samples were taken over several visits.

When stationary measurements were made in new occupational environments, e.g. restaurants and mat welding, where it was unknown if ICA would be generated, samplers were placed close (within 1 m) to the hypothesised source/s to see if any emissions could be quantified at all. For measurements where ICA emission is known to occur, measurements were made at representative places where work was carried out, rather than automatically nearest to the emission source.

### Sampled environments

Measurements were performed at five different restaurants, three food industries, two manufacturers using urea at high temperature and three users of such material, two different forms of thermal decomposition of polyurethane, welding in linoleum carpet, diathermy treatment, smoking, three different engine emission measurements and urban air.

A detailed description of the different environments can be found in the supplement. Briefly, the restaurants were smaller or larger lunch restaurants, a hamburger restaurant, a food truck and also a home kitchen. The food industries included one refining vegetable products, one maker of industrial goods and a coffee roastery. The users of urea at high temperatures manufactured wooden laminate products and other forms of building materials. Users of the manufactured materials were two district heating plants using glass wool for insulation. One of the plants had made smaller renovations with change of part of the insulation and the other was a new plant. The third user was a producer of building modules from chipboards. For thermal decomposition of PUR, measurements were made at a large company producing complex products using polyurethane glue and during 3D printing with thermoplastic polyurethane. For engine exhaust, the measurements were performed in an experimental setup as well as during asphalting, using a gasoline-powered lawn mower, and in three different traffic environments. For smoking, an experimental setup was used to assess the exposure to a home care staff exposed to passive smoking and direct smoke was also measured.

## Results and discussion

Overall, the performed measurements give some information about exposures and emissions from ICA. The majority of the measurements were performed during the COVID-19 pandemic, which made it more difficult than anticipated to perform personal exposure measurements. In total, 173 measurements were performed in 16 different environments. Results have been split up into old “known” environments (Table 1) and new environments (Table 2). All individual results are presented in supplemental table S1.

**Table 1:** Air sampling results for ICA in environments where ICA emission is known to occur

	Personal sampling						Stationary sampling					
Environment	*N*	Measurement method	Measurement time (min)	% above detection limit	Mean concentration µg/m^3^	Max concentration µg/m^3^	*N*	Measurement method	Measurement time (min)	% above detection limit	Mean concentration µg/m^3^	Max concentration µg/m^3^
Cigarette smoke												
*Ambient air (side smoke*)	-	-	-	-	-	-	5	Denuder/impinger	10	100	2.5	5.5
*Direct (smoker’s exposure*)	-	-	-	-	-	-	3	Denuder/impinger	1–2	100	2050	4300
Thermal processing polyurethane	5	Denuder	85–108	80	3.3	4.6	2	Denuder	111,124	50	-	4.1
Heating insulation old	-	-	-	-	-	-	3	Denuder	113–114	0	-	< 0.53*
Heating insulation new	-	-	-	-	-	-	18	Denuder	113–137	100	7.4	64
Manufacturing using urea	10	Denuder	129–142	100	29	94	51	Denuder	105–137	100	58	270
Using chip boards with urea glue	2	Impinger	5	100	0.37	0.47	2	Impinger	5	100	0.33	0.48
Diesel exhaust	**-**	-	-	-	-	-	19	Denuder	16–250	74	2.8	4.6

*For < values, results have been calculated based on the detection limit of the method and the collected air volume. Where there is large discrepancy in collected volume for the same environment, the lowest value (i.e. that resulting from use of the highest collected volume) is presented

**Table 2:** Air sampling results for ICA in environments where ICA emission has not been previously measured

	Personal sampling					Stationary sampling						
Environment	*N* of samples	Measurement method	Measurement time (min)	% above detection limit	Mean concentration µg/m^3^	Max concentration µg/m^3^	N of samples	Measurement method	Measurement time (min)	% above detection limit	Mean concentration µg/m^3^	Max concentration µg/m^3^
Restaurant/home cooking	3	Denuder	123–179	66	0.45	0.55	11	Denuder	26–185	27	0.24	0.38
Food industry	2	Denuder	76–82	0	-	< 0.55*	13	Denuder	48–117	23	1.1	1.4
Mat welding	1	Denuder	15	0	-	< 4.5*	2	Denuder	11–15	50	4.5	5.8
Asphalting	3	Denuder	197–2013	67	0.44	0.52	-	-	-	-	-	-
Diathermy	3	Denuder	51–151	33	-	0.8	3	Denuder	72–150	33	-	1.1
3D printing with TPU (thermoplastic polyurethane) Inside printer	-	-	-	-	-	-	3	Denuder	53–198	67	2.9	4.4
Outside printer	-	-	-	-	-	-	3	Denuder	49–200	0	-	< 0.02*
Use of gasoline-powered lawn mower	1	Denuder	80	100	-	1.4	-	-	-	-	-	-
Urban air	-	-	-	-	-	-	14	Denuder	305–1289	100	0.12	0.25

*For < values, results have been calculated based on the detection limit of the method and the collected air volume. Where there is large discrepancy in collected volume for the same environment, the lowest value (i.e. that resulting from use of the highest collected volume) is presented

### Known environments

#### Manufacture of material containing urea binders

It is very common in industry to use a mixture of urea and other substances as a binder for materials to be used in later technical applications. These are often highly automated industries with large machines and furnaces and with little staff present. Two companies were surveyed and showed similar emission profiles. At the company producing wooden laminate products, measurements were carried out on two repeated occasions. The first measurement was carried out during the spring/winter and then higher concentrations were measured at most of the measurement points. In the repeated measurements carried out in June, the measured concentrations were lower. Production had a high degree of automation and staff sporadically monitored the processes where emissions were highest. The average personal exposure measurements were 48 µg/m^3^ and maximum concentration was 92 µg/m^3^on the first occasion. Repeated measurments were significantly lower (average 19 µg/m^3^, max 30 µg/m^3^; *n* = 4) but relatively high in comparison with the OEL (18 µg/m^3^). Some of the stationary measurements were extremely high with the highest above 264 µg/m^3^.

For the other company producing building material including using urea at high temperatures, the degree of automation was also extensive. The company was also aware that in some places there were considerable emissions from the process and there it was clearly marked that if you entered these zones, personal protective equipment was mandatory. Measurements carried out in these zones showed the highest exposure. The personal measurements that were carried out can be characterised as a “worst-case” scenario; the average exposure for the personal measurements were 2 µg/m^3^ with 30 µg/m^3^ as a maximum. Those with the highest measured exposures conducted “infrequent work” with the aim of measuring how high the potential exposure could be. Nevertheless, the exposure in this environment must be considered high when compared to the OEL.

The measured levels are in the same magnitude as presented earlier in this kind of environments as Jankowski et al. (2017) had a range between 3 and 573 µg/m^3^ and Westberg et al. (2005) measured exposure between < 4 and 190 µg/m^3^ (average 27 µg/m^3^), showing little has changed over the last 10–20 years in terms of exposure in this environment.

#### Users of building materials containing urea resins

The fact that glass wool and mineral wool are products manufactured with binders containing urea is well known. The manufacturers of these products also state that care should be taken when first heating as irritating gases may be emitted. Mineral wool is used to a very large extent as insulation material in Sweden’s large combined heat and power plants (CHP) and release of ICA during heating of the material is expected. Measurements in the study were made at two CHP plants to investigate concentrations emitted from the insulation materials but also how these emissions would differ between newly installed insulation and during minor renovation of already insulated heating parts.

**Fig. 1:**
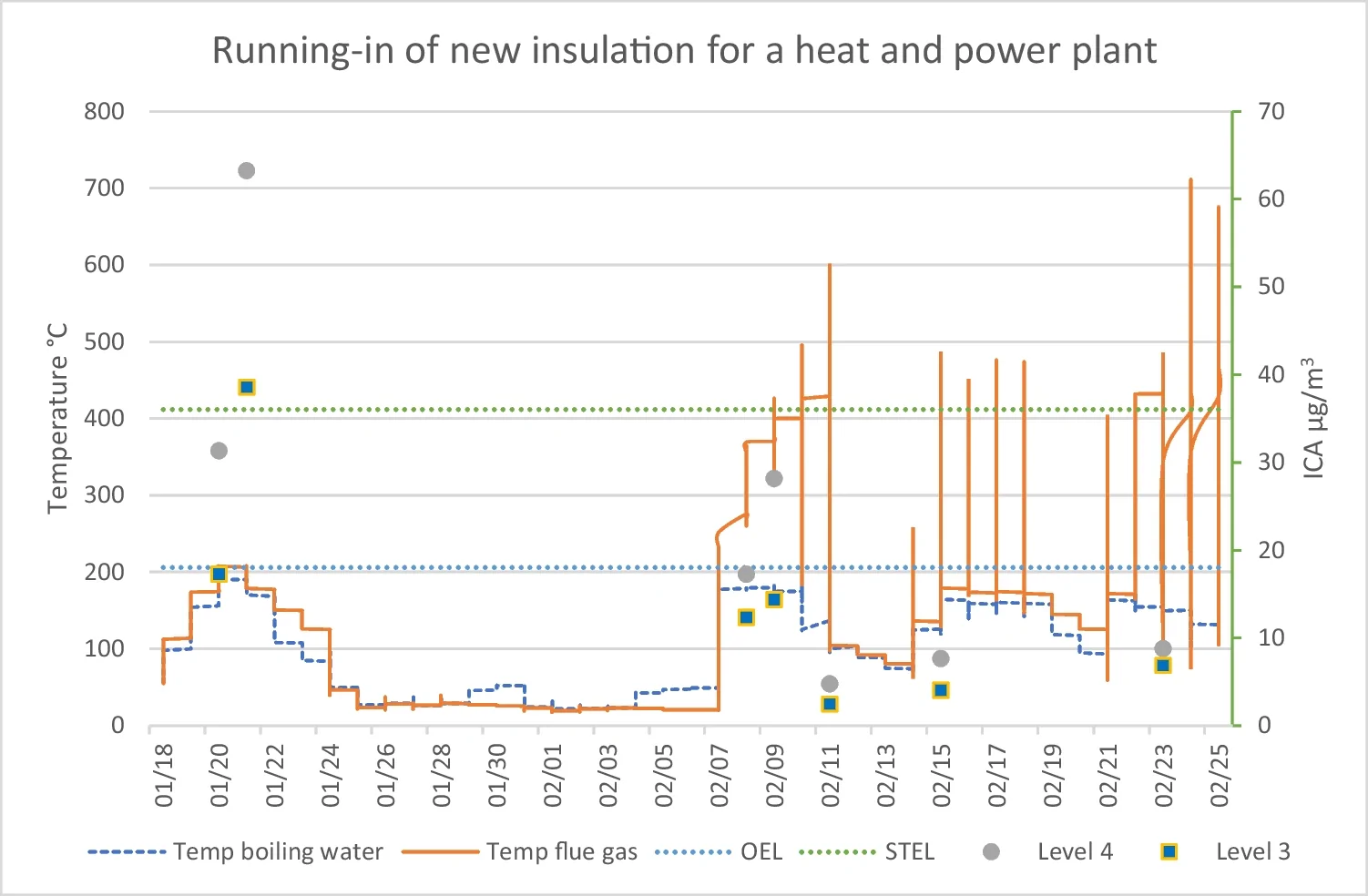
Stationary measurements of ICA concentrations during the running-in period of new insulation at a CHP plant. On the *x*-axis are the dates for the running-in period for the new power plant. On the left *y*-axis is the temperature for the boiling water and for the flue gas. On the right *y*-axis is the level of ICA in µg/m^3^. The circles are measurements on the fourth floor and the boxes the measurements on the third floor. The two dotted lines are the 8-h OEL and the 5-min STEL value

During the minor renovation, no detectable levels of ICA were found. At the brand new facility, measurements were carried out over time, as the running-in temperatures increased and also in different parts of the room (see Fig. 1). From the measurements, it is apparent that certain temperature increases led to higher emissions and, in general, that higher up in the CHP plant there were higher concentrations of ICA (see Fig. 1, measurement point floor 4 always higher than floor 3). The measured levels reached a maximum of 62 µg/m^3^ and remained elevated for a long period after the start-up of the completely new power plant. The highest measured ICA levels were at the first temperature rise over 200 °C. With repeated temperature rises, levels remained high but far from the levels measured during the first warming. This is in accordance with the observations of Jankowski (Jankowski et al. 2016) that the emission of ICA starts at 133 °C, which is the point where urea starts to melt. The operations of both these applications were aware of the risks and there was an edict on the use of personal protective equipment when entering these plants during the start-up phases when there could be an increased risk of exposure from the insulation material.

For the company that used chip boards that were manufactured with a urea-containing binder, measurements were carried out during normal carpentry work. All the samples showed detectable levels, but the measured concentrations were very low (max 0.5 µg/m^3^). The chemical composition of the urea-to-formaldehyde ratio in binders and resins will have an impact on the emission profile of the different materials but this information was not available at the time of measuring.

#### Treatment of polyurethane products with heat

ICA concentrations were measured at two companies that thermally process PUR products. The fact that thermal degradation produces emissions of both diisocyanates and monoisocyanates has been known since the late 1990s (Karlsson et al. 1998). One of the companies, a very large company with several hundred employees, has a hot process involving polyurethane products centrally located in the production hall with a size of about 20,000 m^2^. Close to the emission process, both diisocyanates and monoisocyanates could be detected but further away only ICA had detectable concentrations. This indicates that ICA is not very reactive as it could also be measured far from the source, which fits with the estimated residence time, 8 h, for ICA in air (Jankowski et al. 2016). The company was aware of the problems of emissions spreading to large parts of the premises. In our measurements, concentrations of ICA could be found in the whole building far from the primary source, but levels were below the Swedish OEL. The average personal exposure (*n* = 5) was 3 µg/m^3^, with a max of 5 µg/m^3^.

### Environmental sources

As one of the triggers for this study was the lacking data from environmental measurements of ICA in Europe, measurements were performed in the Gothenburg area at three different locations. At all locations, ICA could be detected, but in very low concentrations. The three sites were chosen with different traffic intensity, but despite this, the measured levels were very similar and were in a small range between 0.14 and 0.25 µg/m^3^, which is in the lower range from earlier presented outdoor data (Leslie et al. 2019) and lower than recent presented data from China with measured levels around 0.7 µg/m^3^ (Wang et al. 2020).

For cigarette smoking, measurements were carried out to investigate the exposure of the smoker but also the exposure of a home care worker if they were with patients who smoked and were exposed to side smoke in the ambient air (i.e. passive exposure). The side smoke exposure was measured at 1–5 µg/m^3^. However, the measured concentrations directly in cigarette smoke were extremely high, up to 4000 µg/m^3^. In the cigarette smoke, we also detected extremely high levels of methyl isocyanate, over 6000 µg/m^3^. These findings are consistent with the literature (Philippe and Honeycutt 1965) and have since been replicated on a larger scale and reach the same conclusion (Runström Eden et al. 2025).

In connection with another project, ICA emissions were measured experimentally at different variants of driving cycles for diesel engines and different diesel fuels. The experimental setup was optimised for the main question of how elemental carbon is emitted at different engines and fuels and not primarily to investigate the emissions of ICA. The measured emissions were also sampled directly from the exhaust pipes, completely without any dilution. There was no clear pattern in how various parameters affected the emissions of ICA and the levels were between 0.5 and 4.6 µg/m^3^. In some internal combustion engines, a diesel exhaust fluid (e.g. AdBlue) that contains urea is added to the exhaust to reduce nitrogen oxide (NOx) emissions. We investigated how this affected the emissions of ICA, but the emissions in those trials were not generally higher than in other runs. It is clear that internal combustion engines are an important source of the measured levels of ICA in urban air.

### New environments

#### Restaurants and food industry

As mentioned earlier, a primary reason for the study was the recent publication by Leanderson that showed high emissions of ICA in certain food handling under experimental conditions. Consequently, measurements were carried out in several types of restaurants and in the food processing industry and the results conclude that the generated ICA concentrations were generally low or very low (max 0.6 µg/m^3^) compared to the more well-known emission sources (e.g. thermal processing of polyurethane or manufacturing using urea, max 4.6 and 270 µg/m^3^ respectively). Only one restaurant, where hamburgers were fried, generated concentrations above the detection limit of the analysis method. During frying at home, all samples showed low emissions, which is in accordance with previous observations with increased levels of ICA indoor (Wang et al. 2022). Subjectively, the ventilation was deemed to be worse in the restaurant with the detectable exposure and in the home environment which may have contributed to the result. Consequently, under normal restaurant conditions with good ventilation, the exposure to ICA is likely very low (no samples had levels above 1 µg/m^3^), but if malfunction of the ventilation occurs, the exposure probably could be higher, according to Leanderson. In the study by Leanderson (2019), where high emissions of ICA were seen, the sampling was performed directly in the fumes 40 cm over the pan and 15 cm below the kitchen fan. In contrast, the present study measured ICA as personal exposure or stationary emissions. Thus, samplers were in close vicinity to the cooking processes but not directly in the fumes. In the food processing industry, five samples were above the detection limit out of a total of 15 samples and these were in close proximity to high-temperature processes. The dwell time of the staff in those areas was very low. The highest measured level was just above 1 µg/m^3^.

#### Welding of linoleum mats

Measurements were made during repair work of linoleum mats. Linoleum is made up of natural materials and repair work often includes welding at high temperatures (300–350 °C), which is why ICA emissions were suspected of being formed. During the measurements, two linoleum mats were welded together with a weld rod and a heating pistol. The measurement carried out directly in the formed smoke showed 6 µg/m^3^, while the personal and background measurements could not detect any concentrations. Thus, it can be stated that a certain level of emission exists, but it was not sufficient to give measurable levels of exposure on a personal level in this case. However, the measurement time was quite short, as the work was short so the detection limit for the personal sample was 4.5 µg/m^3^. Therefore, the situation might be different if there are environments where this work is more frequent.

#### Diathermy

At a dermatology clinic, we measured ICA during diathermy and when using CO_2_ laser. Surgical diathermy involves the use of high-frequency electric currents to cut or coagulate tissue and CO_2_ lasers are used for precise cutting and ablation of tissues. This can lead to the thermal degradation of proteins and other organic compounds in the biological tissues and these processes were hypothesised to result in the formation of ICA due to the breakdown of nitrogen-containing substances. When using CO_2_ lasers, ICA was detected in a personal sample (0.8 µg/m^3^) and a stationary sample (1.1 µg/m^3^) but concentrations were low compared to other environments and the Swedish OEL. No samples above the detection limit were seen for diathermy. A likely cause for this discrepancy is the difference in temperature between the two techniques (ca 100 °C and 400 °C for diathermy and CO_2_ laser respectively).

#### Asphalt work

In asphalt work, workers are close to both asphalt smoke and exhaust fumes from the vehicles used, which gives rise to two possible emission sources. Measurements on the two people working at street level showed detectable concentrations of ICA, but at very low levels (maximum 0.5 µg/m^3^). The exposure for the driver of the asphalt machine was below the detection limit. This indicates that asphalt workers are exposed to ICA but that concentrations are similar to or slightly above background environmental urban air measurements in Gothenburg.

#### Use of gasoline-powered lawn mower

A personal measurement was also carried out mowing the lawn in an ordinary villa garden with a petrol-powered lawn mower and the exposure was just above 1 µg/m^3^. There are several occupations that are exposed to emissions from petrol or diesel engines and whilst very limited, this result in line with the emissions found from diesel engines and low in relationship to the Swedish OEL.

#### Additive manufacturing

Additive manufacturing, which includes 3D printing, is a technology that is increasingly used in industry. During 3D printing using fused filament fabrication, a thin plastic filament is heated up to a temperature where it becomes soft and pliable and this results in release of volatile organic compounds and nanoparticles (Stefaniak et al. 2021). In a previous study, Runström et al. (2022) showed that monoisocyanates can be emitted during printing with thermoplastic polyurethane (TPU) filament. In the measurements in this study, printing was conducted in printers with ventilated hoods. Samplers were placed inside the printer hood and outside the printer, where the operator would be stationed, in two different printer brands and included printing with three different TPU filaments in total. Results confirmed that concentrations of ICA were formed inside the one printer hood for both used TPU filaments, but no concentrations were detected outside the printer. Conversely for the third printer and filament type, emissions were below the detection limit both inside the printer hood and outside of the printer. More studies on this need to be conducted to understand what factors drive the emissions.

### Limitations

There are several limitations to the study. Whilst samples in most cases have been taken from more than one environment handling similar materials, some environments have only been surveyed once (diathermy, asphalting, lawn mowing), which makes the results more uncertain for those tasks. Similarly, there has been variation in sampling time and other factors (e.g. room size, ventilation, processing temperatures, distance from emission source) that could affect measured concentrations. This was expected from the study design as the aim of the study was to survey environments to compare relative concentrations and identify new sources, rather than conduct robust exposure measurements. Similarly, the measured concentrations are compared to the applicable OEL and STEL, whilst samples have not been taken for the required time. The comparison should therefore be used to aid the ranking of the concentrations in the different environments rather than to determine whether the exposure level has been exceeded or not. The denuder samplers used also have a limitation in that they cannot be used for a whole working day. In a few environments, both impingers and denuders have been used, which can introduce additional variation, but laboratory studies have shown that the methods generally follow each other well (Marand et al. 2005) and for the aim of the study this was found to be acceptable.

## Conclusions

There is an obvious gap of information about negative health effects after exposure to ICA and several researchers point out the need for this knowledge (Leslie et al. 2019, Leandersson 2019, Wang et al. 2022). This is not resolved in the current study but the information is desired to better understand the implications of exposure. The overall environmental exposure to ICA is low but, in principle, everyone can expect to be exposed to ICA at 0.2 µg/m^3^ outdoors or a little higher indoors (Wang et al. 2022). Environmental exposure in Gothenburg was in the lower part of the range of previously reported data and these are the first such values reported from Europe. The occupational exposure to ICA is, however, much higher, as presented here and in previous studies (Jankowski et al. 2016, Westerberg et al. 2005).

Of the environments in which measurements have been carried out, exposure was evident in the operations that use urea as a binder at high temperatures, as well as when heating such products to high temperatures for the first time (in this study, mineral wool). A positive aspect is that those businesses were aware that exposure could be considerable and had in several cases taken measures to minimise exposure. For example, during start-up periods where insulation has been changed, the use of personal protective equipment was imposed for heating plants. At the manufacturing units using urea, a lot of work had been done with automation, so that staff were in exposed areas for as short a time as possible. If the same awareness of this exposure is present outside of Sweden, where no OEL exists, is not known to us. On the other hand, in these industrial environments, there are existing co-exposures to other substances, such as formaldehyde, that trigger a precautionary work procedure. Smoking also contributes to a high exposure to ICA and smoking one cigarette provides an exposure above the Swedish 8-h OEL as is further discussed in Runström Eden et al. (2025).

Emissions and exposures from ICA surveyed in new occupational environments showed consistently low or undetectable concentrations (maximum 5.8 µg/m^3^ for linoleum welding). A limitation of this study is the small number of samples taken in each environment. This was due to prioritising the inclusion of a wider variety of environments rather than performing a more detailed survey of fewer environments. Consequently, the results are to be taken with caution but indicate that the predominant risks for high ICA exposure are in the environments where PUR or urea is handled at higher temperatures and during cigarette smoking.

## Data Availability

The authors declare that the data supporting the findings of this study are available within the paper and its supplementary information files. Should any raw data files be needed in another format, they are available from the corresponding author upon reasonable request.
